# Biophysical Characterization of G-Quadruplex Recognition in the PITX1 mRNA by the Specificity Domain of the Helicase RHAU

**DOI:** 10.1371/journal.pone.0144510

**Published:** 2015-12-09

**Authors:** Emmanuel O. Ariyo, Evan P. Booy, Trushar R. Patel, Edis Dzananovic, Ewan K. McRae, Markus Meier, Kevin McEleney, Jorg Stetefeld, Sean A. McKenna

**Affiliations:** 1 Department of Chemistry, University of Manitoba, 144 Dysart Road, Winnipeg, Manitoba R3T 2N2, Canada; 2 School of Biosciences, University of Birmingham, Birmingham B152TT, United Kingdom; 3 Manitoba Institute for Materials, University of Manitoba, 144 Dysart Road, Winnipeg, Manitoba R3T 2N2, Canada; 4 Department of Biochemistry and Medical Genetics, University of Manitoba, 144 Dysart Road, Winnipeg, Manitoba R3T 2N2, Canada; University of Quebec at Trois-Rivieres, CANADA

## Abstract

Nucleic acids rich in guanine are able to fold into unique structures known as G-quadruplexes. G-quadruplexes consist of four tracts of guanylates arranged in parallel or antiparallel strands that are aligned in stacked G-quartet planes. The structure is further stabilized by Hoogsteen hydrogen bonds and monovalent cations centered between the planes. RHAU (RNA helicase associated with AU-rich element) is a member of the ATP-dependent DExH/D family of RNA helicases and can bind and resolve G-quadruplexes. RHAU contains a core helicase domain with an N-terminal extension that enables recognition and full binding affinity to RNA and DNA G-quadruplexes. PITX1, a member of the bicoid class of homeobox proteins, is a transcriptional activator active during development of vertebrates, chiefly in the anterior pituitary gland and several other organs. We have previously demonstrated that RHAU regulates PITX1 levels through interaction with G-quadruplexes at the 3’-end of the PITX1 mRNA. To understand the structural basis of G-quadruplex recognition by RHAU, we characterize a purified minimal PITX1 G-quadruplex using a variety of biophysical techniques including electrophoretic mobility shift assays, UV-VIS spectroscopy, circular dichroism, dynamic light scattering, small angle X-ray scattering and nuclear magnetic resonance spectroscopy. Our biophysical analysis provides evidence that the RNA G-quadruplex, but not its DNA counterpart, can adopt a parallel orientation, and that only the RNA can interact with N-terminal domain of RHAU via the tetrad face of the G-quadruplex. This work extends our insight into how the N-terminal region of RHAU recognizes parallel G-quadruplexes.

## Introduction

G-quadruplexes (G4) are four-stranded structures of DNA or RNA in which one guanine base from each chain associates via cyclic Hoogsteen [1] hydrogen bonding to form planar quartets. Two or more such quartets hydrophobically stack on top of each other to form the G4 and are stabilized by the presence of a mandatory monovalent cation (typically K^+^) in the center between the planes [2]. G4s in DNA and RNA can adopt a parallel, anti-parallel, or hybrid (mixture of both parallel and antiparallel) strand orientation [3]. Biophysical studies and high-resolution structures of RNA G4s reveal that they are thermodynamically more stable *in vitro* than their DNA counterparts under near-physiological conditions because of the 2′-OH on the ribose sugar that permits additional hydrogen bonds to form. As a result, RNA G4s preferentially adopt a parallel conformation over an antiparallel one [4–6].

A survey of the evolutionary conservation of DNA and RNA motifs revealed that G4 motifs are significantly conserved in the genomes of living organisms [7–10], and it was recently demonstrated that G4 formation is regulated dynamically during cell-cycle progression [11, 12]. Accumulating evidence suggests an important role of G4 structures in regulating gene expression [10]. Genome-wide computational analysis has identified more than 300,000 potential intramolecular G4-forming sequences in the human genome [9, 13] and revealed a higher prevalence of these sequences in functional genomic regions such as telomeres, promoters [10, 14], untranslated regions (UTRs) [15, 16] and introns [17]. Taken together, these observations suggest that G4 structures participate in regulating myriad biological processes.

DNA G4 recognition and remodeling by helicases such as Fanconi anaemia group J protein (FANCJ), Bloom syndrome protein (BLM), DNA repair protein (REV1) and Werner’s syndrome protein (WRN) have been reported [18–21]. RNA Helicase Associated with AU-rich element (RHAU, DHX36, G4R1) is a member of the human ATP-dependent DEAH-box family of RNA helicases, although DNA G4 helicase activity has also been observed with this enzyme [22, 23]. RHAU uses a local, non-processive mechanism to unwind G4s, similar to that of eukaryotic initiation factor 4A on double-stranded substrates [24, 25]. RHAU has nanomolar to sub-nanomolar affinity for G4s, and orders of magnitude weaker affinity for other observed nucleic acid conformations [26–31]. Furthermore, RHAU has 100-fold higher affinity for parallel relative to non-parallel G4s [24, 28, 30]. Helicase activity is highly sensitive to G4 stability, with an inverse correlation observed [24]. Based on domain conservation with other helicases, RHAU’s core DEAH-box helicase domain (residues 210–614) is flanked by an N-terminal G4-recognition domain (residues 1–210) and C-terminal helicase associated domains (residues 670–1008) that have yet to be fully characterized [32]. G4 specificity is mediated by the RHAU-specific motif (RSM), a 13-residue stretch (residues 54–66) in an N-terminal subdomain that is necessary, but not sufficient for full G4 binding affinity [27, 33]. A truncation of the full-length protein, RHAU_53-105_, adopts a defined and extended conformation in solution, orienting the RSM at one end [31]. RHAU_53-105_ retains both nanomolar affinity for G4s *in vitro* and the ability to outcompete endogenous RHAU for G4 targets in a cellular context [26, 31]. Investigation of RHAU_53-105_ in complex with RNA G4 from human telomerase RNA by NMR, SAXS, and complimentary biophysical approaches suggested interaction with the G-tetrad face (as opposed to the sugar-phosphate backbone) as the recognition surface for the RSM [31]. This mode of recognition was partially supported by a recent high-resolution structure of an 18 amino acid peptide (RHAU_53-70_) in complex with a parallel DNA G4 showing 4 hydrophobic amino acids (G59, I62, G63, A67) mediating interaction on the G-tetrad faces [30]. While the structure also suggests electrostatic interactions with the phosphate backbone, a previous study demonstrated no significant impact of charged amino acid mutations in the RSM [33]. Differences between DNA and RNA G4 binding by RHAU have also previously been reported, likely owing to the conformational restraints imposed by the 2’-OH in RNA [28, 30, 31].

To expand our understanding of biologically relevant RNA G4 recognition by the helicase RHAU, we previously performed an RNA co-immunoprecipitation screen and identified the messenger RNA (mRNA) for the protein Pituitary homeobox 1 (PITX1, P-OTX, backfoot) [26]. PITX1 functions as a transcription factor that plays a pivotal role in the differentiation of the developing pituitary gland, craniofacial structures and hind limbs in early embryonal development [34–37]. Recently, malformations in the lower limbs could be attributed to mutations in the PITX gene [38]. Deletions in PITX1 cause a spectrum of lower-limb malformations including mirror image polydactyly. PITX1 expression is down regulated in a number of tumor types including lung, colorectal, gastric and esophageal cancer and reduced PITX1 expression has been correlated with decreased overall patient survival [39–41]. Most interestingly, the PITX1 mRNA possesses three distinct G4 forming sequences in the 3′-untranslated region (UTR) of its mRNA (Q1: PITX1_1371-1400_, Q2: PITX1_1901-1930_, and Q3: PITX1_2044-2079_). These G4s play roles in the recruitment of RHAU to the PITX1 mRNA and ultimately regulate PITX1 protein translation [26]. In cell lysates and with purified components, both RHAU and RHAU_53-105_ can interact with Q1, Q2, or Q3. Here, we characterize the Q2RNA/RHAU_53-105_ complex using a combination of electrophoretic mobility shift assays, UV-VIS spectroscopy, circular dichroism, dynamic light scattering, small angle X-ray scattering (SAXS) and nuclear magnetic resonance spectroscopy. Our integrated approach suggests that the RSM recognizes the planar guanine quartet face of parallel RNA G4s.

## Materials and Methods

### G4 preparation

All synthetic RNA and DNA were ordered from Integrated DNA Technologies (Coralville, Iowa), and were certified by the manufacturer by mass spectrometry and provided desalted. PITX1_1901-1930_ RNA (Q2RNA) and PITX1_1901-1930_ DNA (Q2DNA) were dissolved in 10 mM Tris (pH 7.5), 100 mM KCl, 1 mM EDTA at a concentration of 5 μM. Samples were heated to 95°C for 5 min and cooled slowly to room temperature to form G4. Nucleic acid in conformations other than G4 were then removed by size exclusion chromatography (SEC) on a HiLoad Superdex 75 26/60 in 10 mM Tris (pH 7.5), 100 mM KCl, and 1 mM EDTA (ÄKTA, GE Healthcare) as described previously (5 mL load volume) [42]. Sample purity was confirmed by both native and denaturing gel electrophoresis separately (15% native or denaturing Tris/Borate/EDTA polyacrylamide gels using urea as a denaturant). Quadruplex-forming nucleic acids were detected with *N*-methyl mesoporphyrin IX (NMM) staining (Frontier Scientific, Logan, UT, USA). Extinction coefficients (260 nm) were calculated from the sequence using IDT SciTools (OligoAnalyzer3.1, Integrated DNA Technologies) and corrected for hyperchromicity using the absorption spectra at 20°C and 90°C: Q2RNA, 253550 M^-1^cm^-1^; Q2DNA 264200 M^-1^cm^-1^.

### Protein expression and purification

RHAU_53-105_ and full length RHAU were expressed and purified as described previously [31, 43]. After removal of the hexahistidine affinity tag by thrombin digestion, the protein was further purified by size exclusion chromatography on a HiLoad Superdex 75 26/60 (ÄKTA GE Healthcare, Mississauga, Canada) in 10 mM HEPES (pH 7.5), 150 mM NaCl (5 mL load volume). Isotopically enriched ^15^N-labelled RHAU_53-105_ was overexpressed in M9 minimal medium according to the method described previously [31]. The extinction coefficient (7020 M^-1^cm^-1^) was used to determine the protein concentration by measuring absorbance at 280 nm, and confirmed by Bradford assay.

### G4-protein complex preparation

G4s and RHAU_53-105_ were diluted to 10 μM in the corresponding buffers described above, mixed in an equimolar ratio, and agitated slowly on a rotator for 15 min at room temperature. Complexes were separated from individual components by SEC on a HiLoad Superdex 75 26/60 column (GE-Healthcare, Mississauga, Canada) for purification in 10 mM Tris (pH 7.5), 100 mM KCl, and 1 mM EDTA. Both protein and nucleic acid components in the complex were confirmed by gel electrophoresis following complex purification. The concentration of the complex was determined by UV absorption using the extinction coefficient ε_260nm_ of Q2 RNA, since the nucleic acid dominates the spectrum.

### Electrophoretic mobility shift assays (EMSA)

EMSAs were performed by combining RNA (150 nM) with increasing concentrations of RHAU or RHAU_53-105_ (0–700 nM) in 50 mM Tris-acetate (pH7.8), 100 mM KCl, 10 mM NaCl, 3 mM MgCl_2_, 70 mM glycine, 10% glycerol, and incubating them at room temperature for 15 minutes. RNA-protein complexes were resolved by native 15% polyacrylamide gels (29:1 acrylamide:*bis* ratio) in 0.5x Tris/Borate/EDTA (TBE) at 80 V, 4°C for 2 hours. After electrophoresis, gels were stained with SYBR Gold fluorescent nucleic acid dye (Invitrogen, Burlington, ON), and imaged on a Fluorchem Q imager using Cy2 excitation LEDs and emission filters (ProteinSimple, San Jose, California). Bands were quantified from three independent experiments using AlphaView-FlorChem Q software provided by the manufacturer.

### Microscale thermophoresis (MST)

Binding reactions were prepared in 50mM Tris-HCl buffer (pH 7.8) with 150 mM NaCl, 10 mM MgCl_2_, 0.5% Glycerol and 0.05% Tween to a total volume of 20 μL. RHAU_53-105_ was diluted 16 times by 2:1 serial dilution to achieve concentrations ranging from 250–0.6nM, and mixed with fluorescent 3’-FAM labeled Q2RNA (purchased from Integrated DNA Technologies, Coralville, Iowa) was held constant at 25 nM. Premium coated capillaries (NanoTemper Technologies, San Francisco, CA) were used for all measurements. Measurements were performed at an LED power 90% and MST-IR power 40% on the Monolith NT.115 instrument under room temperature conditions (21.5°C). For each run the infrared laser was applied for 35 seconds and the reverse T-Jump data signals of the MST-traces were fitted using the law of mass action for 1:1 binding to obtain *K*_*D*_ values.

### Dynamic Light Scattering (DLS)

DLS data were collected on a Nano-S Dynamic Light Scattering system (Malvern Instruments Ltd., Malvern, UK) as previously reported [44]. Samples were filtered through a 0.1-μm filter (Millipore) and equilibrated for 5 minutes at 20°C before measurements. 15 measurements were made per sample, and for each condition three independent samples were tested.

### Thermal difference spectra

UV/VIS spectra were obtained on a dual beam Evolution 260 Bio UV-Visible spectrophotometer (Thermo Scientific). Q2RNA and Q2DNA (2 μM) in 10 mM Tris (pH 7.5), 100 mM KCl, and 1 mM EDTA were measured in triplicate and background corrected against spectra of buffer alone. Thermal difference spectra (TDS) were generated by subtracting buffer-corrected spectra at 20°C from those at 90°C. For direct comparison between Q2RNA and Q2DNA, differences were normalized to the maximum observed absorbance value, as previously suggested by Mergny *et*. *al*. [45].

### Circular dichroism spectropolarimetry (CD)

All spectra were recorded on a calibrated Alfa Aesar J-810 spectropolarimeter (Jasco Inc., USA) from 200–340 nm in a 1.0 mm cell and a 32 s integration time. Sample concentrations were kept at 20 μM in 10 mM Tris (pH 7.5), 100 mM KCl, 1 mM EDTA. Measurements were performed in triplicate and baseline-corrected by subtraction of the buffer alone. Circular dichroism thermal melting curves were generated in the same buffer, following the ellipticity at 262 nm with spectra normalized by the number of nucleotides (glycosidic bonds) per unit volume.

### Small angle X-ray scattering (SAXS)

SAXS data were collected using a Rigaku 3-pinhole camera (S-MAX3000) equipped with a Rigaku MicroMax + 002 microfocus sealed tube (Cu-Kα radiation at 1.54 Å) and Confocal Max-Flux (CMF) optics operating at 40 W as previously reported [46]. Scattering data were collected at the following sample concentrations; Q2RNA and Q2DNA (0.8, 1.1, and 1.7 mg/ml), and Q2RNA/RHAU_53-105_ complex (1.3 and 1.5 mg/mL) in 10 mM Tris (pH 7.5), 100 mM KCl, 1 mM EDTA. The raw intensity data were integrated with the SAXSGUI software package (JJ X-Ray Systems A/S, LyngBy, Denmark). Buffer subtraction and merging of data of multiple concentrations were performed using the program PRIMUS [47]. The pair distance distribution function plot, root mean square radius of gyration (*r*_*G*_) and the maximum particle dimension (*D*_*max*_) were obtained using the program GNOM [48]. *Ab initio* shape modeling was performed using the program DAMMIF based on a simulated annealing protocol [49, 50]. Twenty models for each entity were then generated, rotated, aligned and averaged using the program DAMAVER [51]. HYDROPRO [52] was used to calculate solution hydrodynamic properties of the averaged-filtered models using a similar approach as outlined previously [46]. Sample quality was confirmed for each sample before and after data collection by gel electrophoresis and DLS.

### Nuclear magnetic resonance (NMR) spectroscopy

^15^N RHAU_53-105_ in complex with Q2RNA (85 μM) in 10 mM Tris, (pH 7.5), 100 mM KCl, 1 mM EDTA, and 10% deuterium (v/v) was prepared in an identical manner as previously described [31]. All spectra were acquired on a Varian Unity INOVA 600 MHz spectrometer. Data processing and spectrum generation were performed using iNMR (http://www.inmr.net).

## Results

### Q2RNA and Q2DNA each adopt a single, monomeric conformation

**Fig 1:**
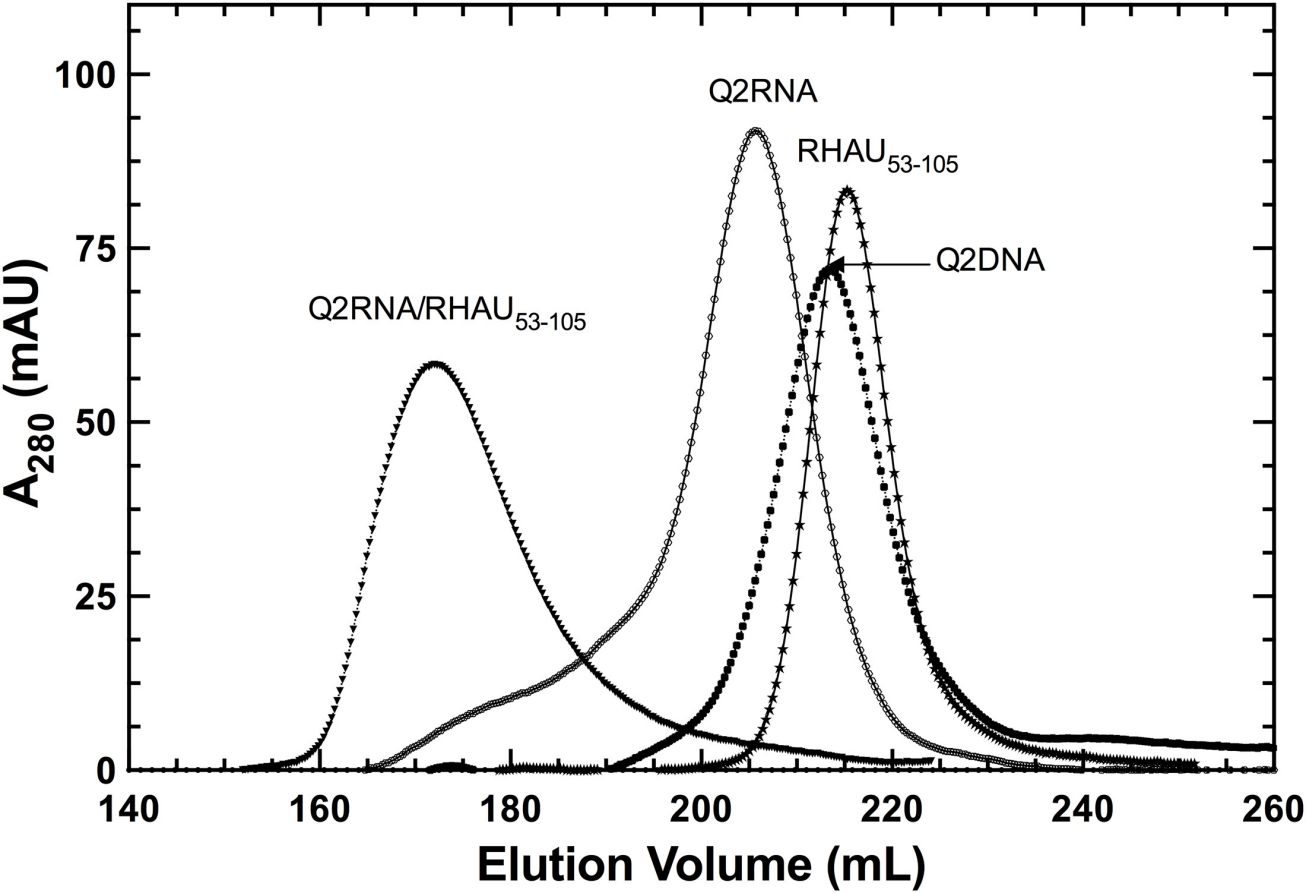
Purification of protein and nucleic acid components. Elution profiles obtained on a HiLoad Superdex 75 26/60 column, with each species labeled.

**Fig 2:**
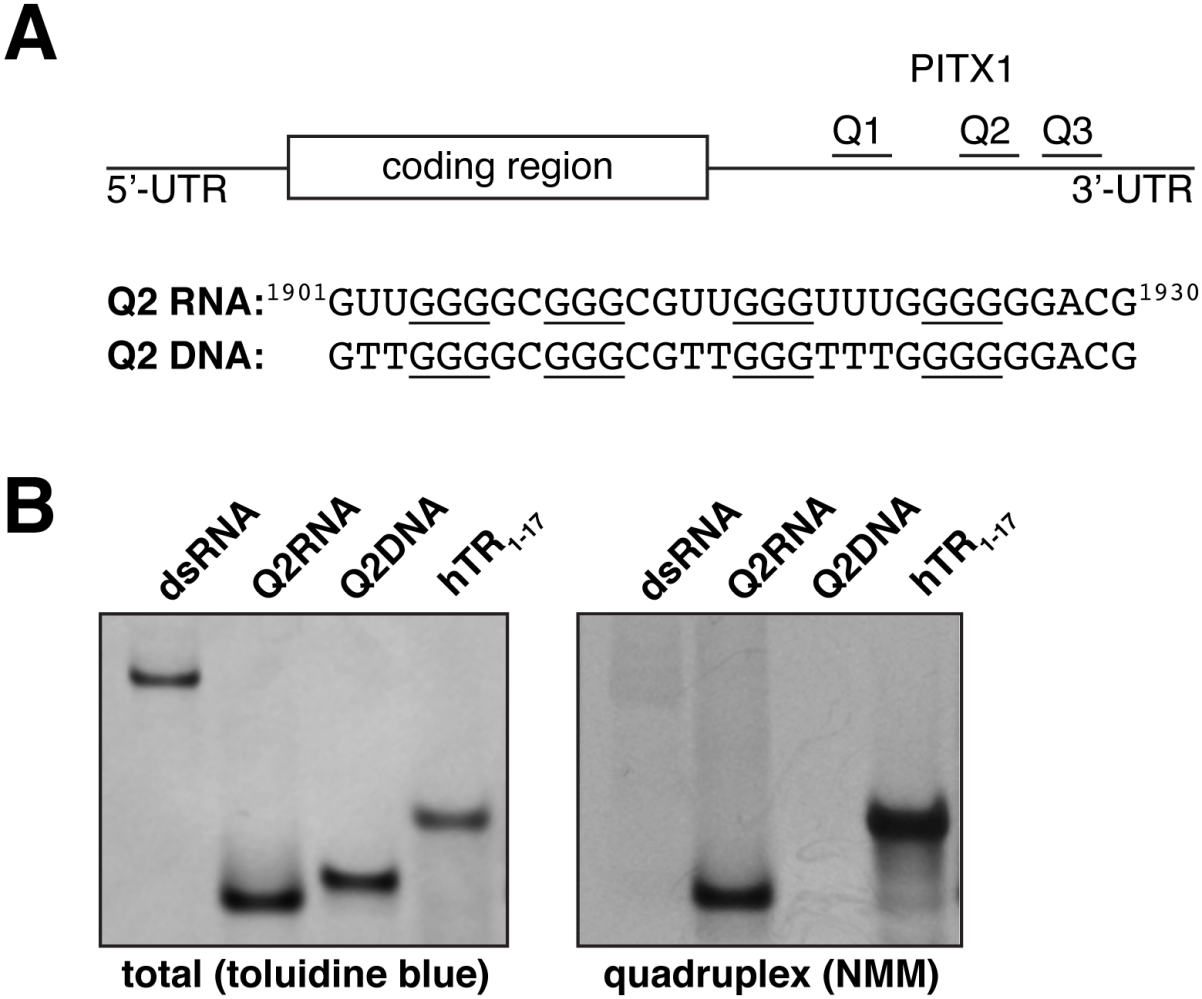
Q2RNA, but not Q2DNA, stains with a parallel G4 dye. (A) Schematic showing the G4 forming regions of PITX1 mRNA; the RNA and DNA equivalent sequences with the guanylate tracts underlined are also shown. 250 pmol of Q2RNA and Q2DNA were separated by native-Tris-borate EDTA (TBE) polyacrylamide gel electrophoresis, stained with (B) toluidine blue, and (C) G4-specific dye *N*-methyl mesoporphyrin IX alongside their positive (G4: hTR_1-17_) and negative (double stranded RNA) controls.

Synthetic Q2RNA was heat denatured, cooled, and purified by size exclusion chromatography (see Materials and Methods). Q2RNA elutes as a compact dominant peak with a shoulder corresponding to larger hydrodynamic volumes (Fig 1) from the HiLoad Superdex 75 26/60 column. The nucleic acid sequences used in this study are presented in (Fig 2A). Native gel electrophoresis confirmed that the dominant peak contains a single RNA conformation (Fig 2B). To understand the potential differences between RNA and DNA G4 recognition, we also investigated the DNA equivalent to Q2RNA (Q2DNA). Using an identical procedure, Q2DNA eluted in a single symmetric peak that contains a single conformation as determined by native gel electrophoresis (Figs 1 and 2B).

### Q2RNA, but not Q2DNA, stains with a dye specific for parallel G4

To determine whether the purified nucleic acids adopt a parallel G4 conformation, we employed native gel electrophoresis in combination with *N*-methyl mesoporphyrin IX (NMM), a dye specific to parallel G4 conformations. The crystal structure of NMM bound to a parallel DNA G4 demonstrates the selectivity for parallel G4s [53]. To confirm the validity of the approach, non-G4 double-stranded RNA (dsRNA) and a known parallel RNA G4 (hTR_1-17_) were included as negative and positive controls, respectively. While both stain efficiently with toluidine blue (nucleic acid stain, pH 2.8), the positive, but not negative control stains with NMM (Fig 2B and 2C). Q2RNA stains efficiently with NMM, indicating that it adopts a parallel G4 conformation. Interestingly, the Q2DNA does not stain with the dye, suggesting that either it adopts a non-G4 or non-parallel G4 conformation.

### Q2RNA adopts a parallel, while Q2DNA adopts an alternate, G4 conformation

**Fig 3:**
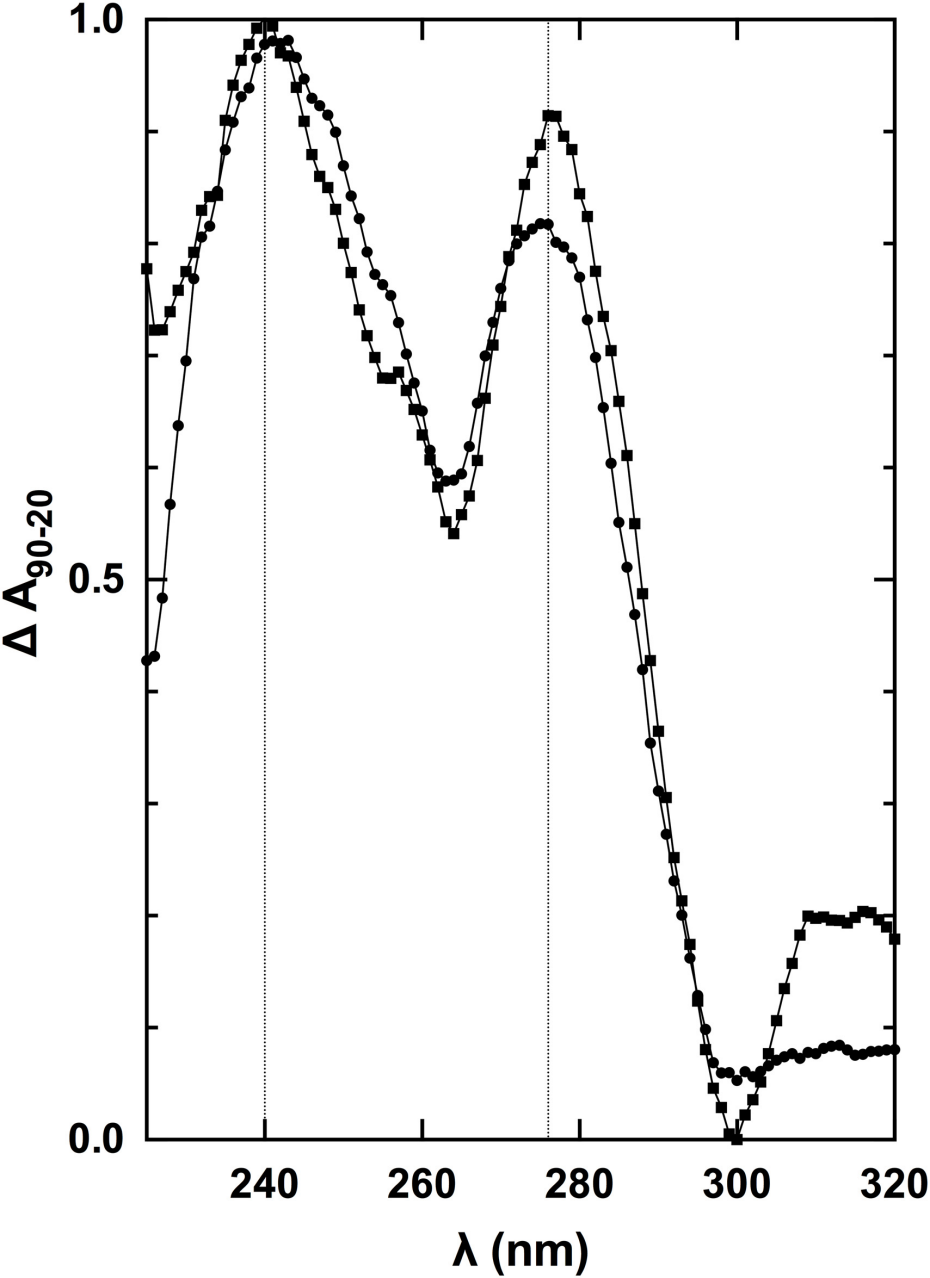
Normalized thermal difference spectra of Q2RNA and DNA counterpart. TDS of Q2RNA (circles) and Q2DNA (squares) in 10 mM Tris, pH 7.5, 100 mM KCl, 1 mM EDTA. For details of analysis, see Materials and Methods.

To confirm G4 formation by Q2RNA, thermal difference spectra (TDS) were generated by subtracting the UV absorption spectrum of the folded state (recorded at 20°C) from the spectrum of the partially denatured state (measured at 90°C). Specific nucleic acid conformations result in specific TDS, generally reflecting the conformational change of the molecule in solution due to a disruption in base-stacking interactions. The TDS obtained for Q2RNA demonstrated features characteristic of G4s [45] with a minimum at 297 nm and two maxima at 240 and 276 nm (Fig 3). Interestingly, TDS analysis of Q2DNA showed similar overall features suggestive that it also adopts a G4 structure.

**Fig 4:**
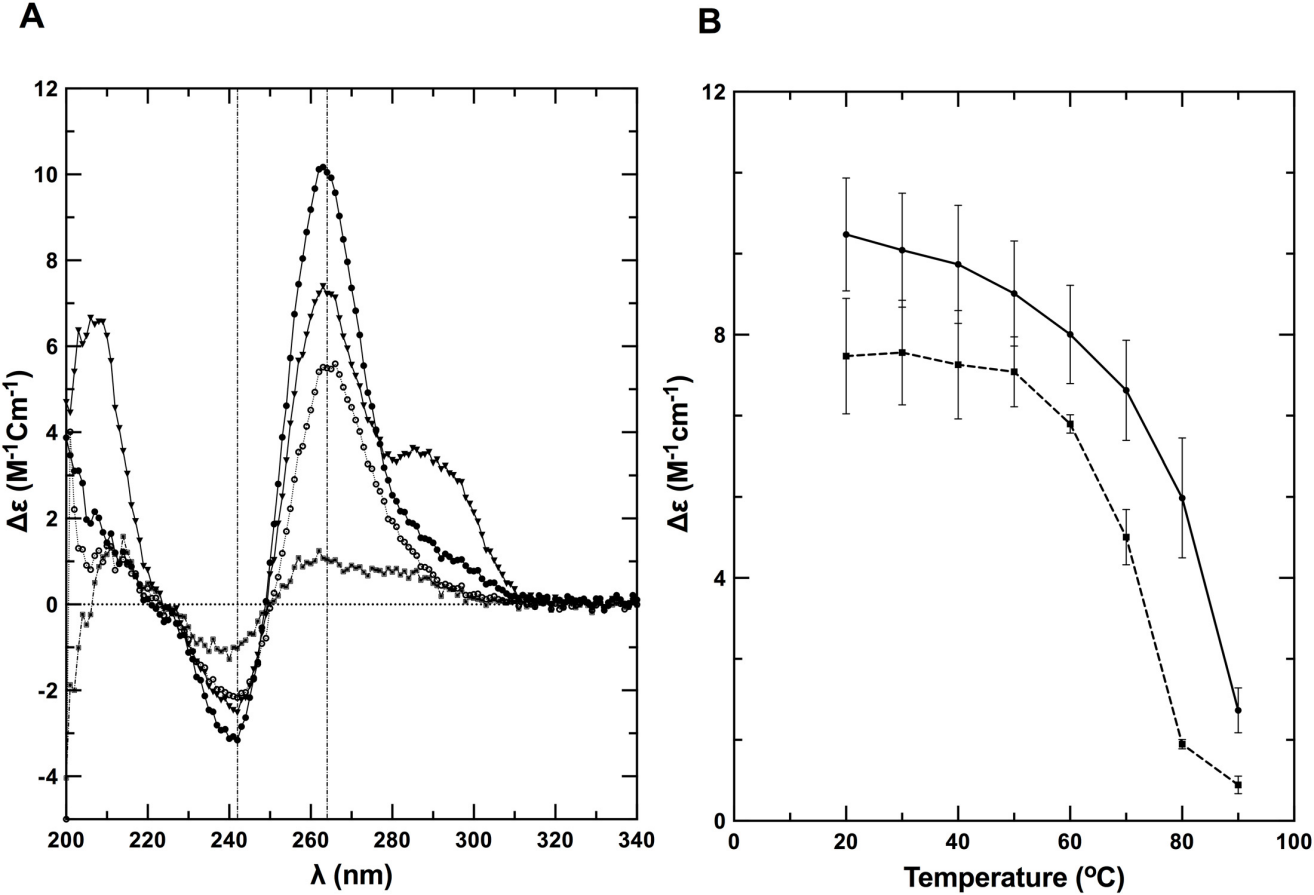
Q2RNA and its DNA counterpart adopt G4 structures. (A) Far-UV CD spectra of Q2RNA and Q2DNA obtained in 10 mM Tris, pH 7.5, 100 mM KCl, 1 mM EDTA buffer of Q2RNA at 20°C (closed circles) and at 80°C (open circles) as well as of Q2DNA at 20°C (closed triangles) and at 80°C (closed squares). (B) CD melting curves of Q2RNA (**——**) and Q2DNA (**------**) monitored by spectropolarimetry at 264 nm in the same buffer.

Next, we performed circular dichroism (CD) spectroscopy on the partially denatured and native states at 80°C and 20°C, respectively, in the same buffer as used for TDS analysis. The far-UV CD spectrum of Q2RNA at 20°C presented features consistent with previously characterized parallel G4s, with an ellipticity minimum at 242 nm and maximum at 264 nm [54] (Fig 4A). At 80°C similar overall spectral features were observed, however the intensity was modestly muted (approximately 45% at 264 nm), presumably due to partial unstacking of the G4 structure. Q2DNA has similar overall features to Q2RNA at 20°C with the prominent exception of an additional maxima at 290 nm (Fig 4A). The Q2DNA spectrum is consistent with the features of a group II G4 spectrum that has three parallel and one antiparallel strands [55–57]. At 80°C, the Q2DNA is almost completely denatured. To determine the relative stabilities of the RNA and DNA G4s, CD spectra were collected during the process of thermal melting (Fig 4B). Q2RNA was significantly more resistant to denaturation than its DNA counterpart, but the melting profile is similar to that of previously characterized RNA G4s [31]. We conclude that Q2RNA adopts, as expected, a parallel G4 conformation, whereas Q2DNA assumes a hybrid-type G4 structure with parallel and antiparallel strands.

### RHAU interacts with Q2RNA but not its DNA counterpart

**Fig 5:**
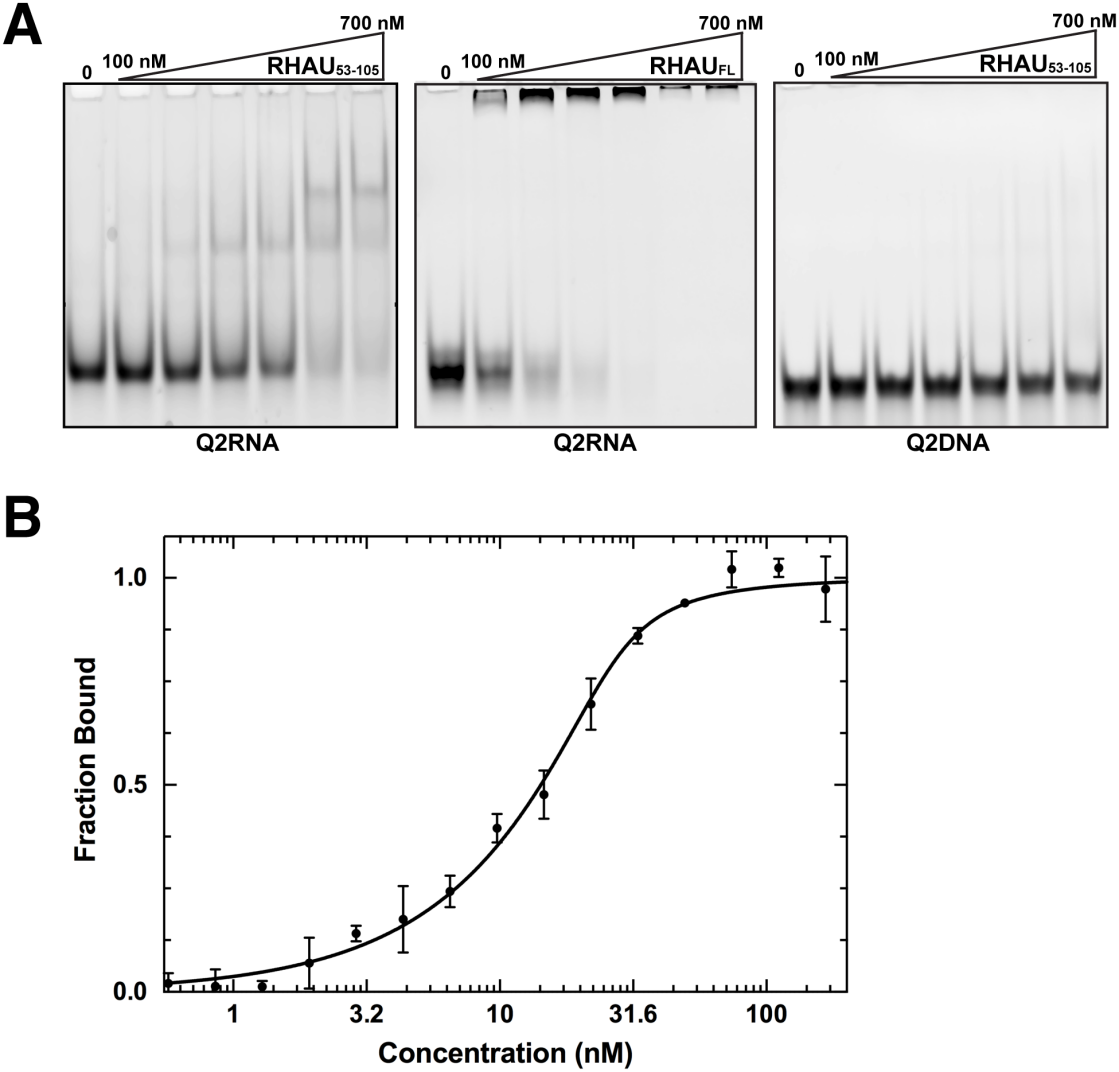
RHAU_53-105_ forms a complex with Q2RNA. (A) Electrophoretic mobility shift assays (EMSA) were performed using a constant 150 nM concentration of Q2RNA or Q2DNA and a variable concentration from 0–700 nM of RHAU_53-105_ or full-length RHAU. The 12% native Tris borate-EDTA (TBE) polyacrylamide gels were stained with SYBR Gold for visualization. (B) Microscale thermophoresis measurements performed using 3’-FAM Q2RNA (25 nM) in complex with RHAU_53-105_ at several concentrations (0.6–250 nM). Reverse T-Jump signals from the traces were fit as described in the Materials & Methods.

Previously, an N-terminal truncation of RHAU (RHAU_53-105_) containing the RSM has been identified to play a significant role in the recognition of G4s [31, 33]. To confirm the original observation, we performed electrophoretic mobility shift assays (EMSA) between Q2RNA and either RHAU_53-105_ or full-length RHAU (Fig 5A). Both RHAU_53-105_ and full-length RHAU shift Q2RNA towards a higher molecular weight species in a concentration dependent manner. We observed a higher affinity with full-length RHAU than with the truncated version (as expected). Interestingly, the DNA counterpart, Q2DNA, did not show any appreciable affinity for RHAU_53-105._ (Fig 5A). Microscale thermophoresis measurements were used to determine a dissociation constant of 1.7±0.3 nM for the RHAU_53-105_ complex with fluorescently labeled 3’-FAM-Q2RNA (Fig 5B).

To further characterize nucleic acid-protein complexes, we prepared pure RHAU_53-105_ as well as its complex with Q2RNA or Q2DNA, and subjected them to size exclusion chromatography (Fig 1). RHAU_53-105_ and its complex with Q2RNA eluted as single peaks, with an expected increase in hydrodynamic size accompanying complex formation. Not surprisingly, no complex formation was observed between Q2DNA and RHAU_53-105_ (data not shown).

### The association of N-terminal RHAU with Q2RNA does not disrupt G4 structure

**Fig 6:**
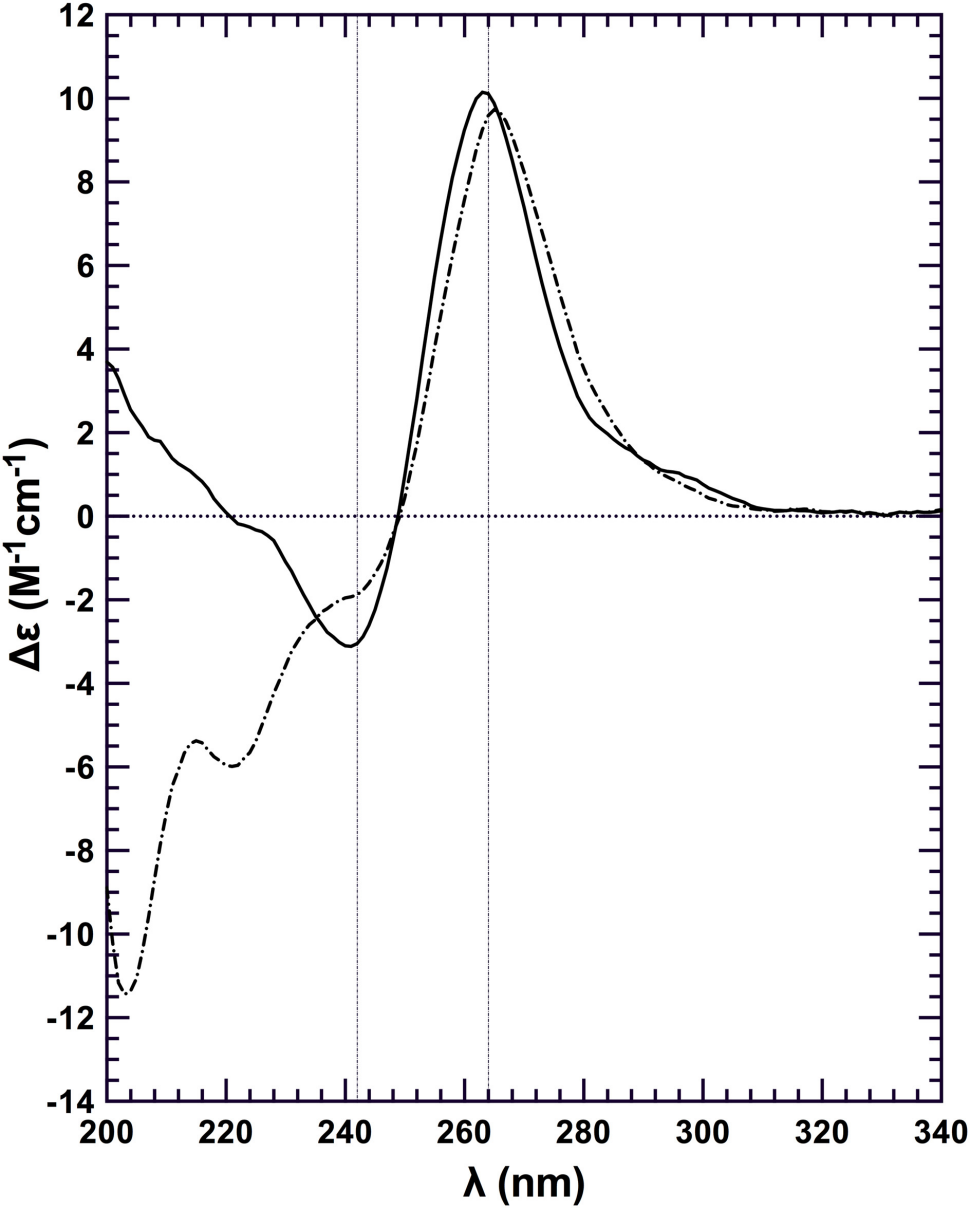
RHAU_53-105_ binding is not sufficient for G4 unwinding. Far-UV CD spectra of Q2RNA (**——**); and Q2RNA/RHAU_53-105_ complex (**··—··**) at 20 μM and 20°C with all the spectra normalized to the number of nucleotides. The G4 features of the RNA in the context of the complex are observed in the region unique to nucleic acids (~250–320 nm).

To determine whether RHAU_53-105_ binding disrupts G4 structure we performed a CD experiment on the purified Q2RNA/RHAU_53-105_ complex (Fig 6). No significant differences were observed between CD spectra from Q2RNA/RHAU_53-105_ and Q2RNA in the region unique to nucleic acids (~250–320 nm), suggesting that the G4 remains intact upon protein binding.

### Solution structures of G4s and their complexes with RHAU_53-105_

**Fig 7:**
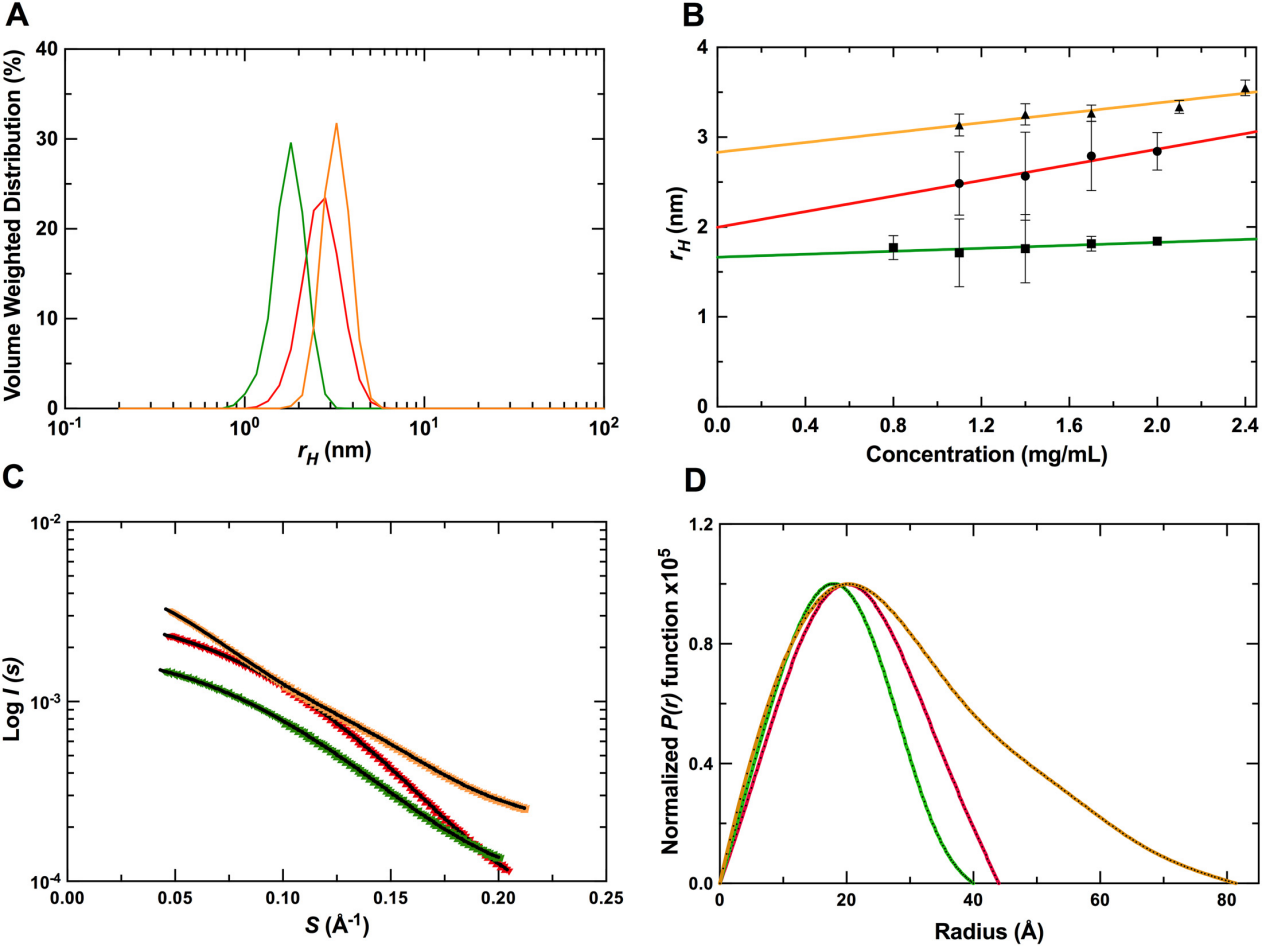
SAXS data collection and analysis. (A) DLS profiles of Q2RNA, Q2DNA and Q2RNA/RHAU_53-105_ complex. (B) Concentration dependence of hydrodynamic radii measured by DLS of the molecules in (A). (C) Merged SAXS data of Q2RNA, Q2DNA and Q2RNA/RHAU_53-105_ complex. (D) The corresponding pair distance distribution functions. (Color code: red, Q2RNA; green, Q2DNA; golden, Q2RNA/RHAU_53-105_ complex).

To further understand the recognition of G4s by RHAU_53-105_, we used SAXS to study Q2RNA, Q2DNA, and the Q2RNA/RHAU_53-105_ complex purified by size exclusion chromatography. DLS was employed as an initial quality control step to ensure sample monodispersity over the range of concentrations used for SAXS acquisition (Fig 7A). Decreasing hydrodynamic radii (*r*_*H*_) were observed for the molecules in the following order: Q2RNA/RHAU_53-105_ complex (3.65 nm), Q2RNA (2.01 nm) and Q2DNA (1.65 nm) (Table 1). Samples did not display any significant self-association in the concentration range subsequently used for SAXS analysis, suggesting suitability for further structural studies (Fig 7B).

**Table 1:** Summary of Hydrodynamic Parameters. Standard deviations are indicated in parentheses, and dashes indicate that the values could not be determined.

	Q2RNA		Q2DNA		Q2RNA/RHAU_53-105_	
Parameter	Exp.	Model^d^	Exp.	Model^d^	Exp.	Model^d^
*r*_*H*_ *(nm)* ^a^	2.01 (0.25)	2.28 (0.01)	1.65 (0.04)	2.03 (0.01)	3.65 (0.10)	2.64 (0.02)
*r*_*G*_ *(nm)* ^b^	1.62 (0.01)	1.81 (0.01)	1.42 (0.02)	1.60 (0.01)	2.36 (0.06)	2.47 (0.01)
*D*_*max*_ *(nm)* ^c^	4.4	4.6 (0.04)	4.0	4.11 (0.03)	8.1	8.2 (0.06)
*χ*	0.53	-	0.82	-	0.80	-
*NSD*	0.5 (0.01)	-	0.5 (0.01)	-	0.63 (0.04)	-

^a^ determined from DLS with error from linear regression analysis to infinite dilution from multiple concentrations.

^b^ determined from SAXS data with error obtained from P(r) analysis by GNOM.

^c^ determined from SAXS data obtained from P(r) analysis by GNOM.

^d^ Model-based parameters calculated from HYDROPRO with errors obtained as the S.D. from multiple models.

**Fig 8:**
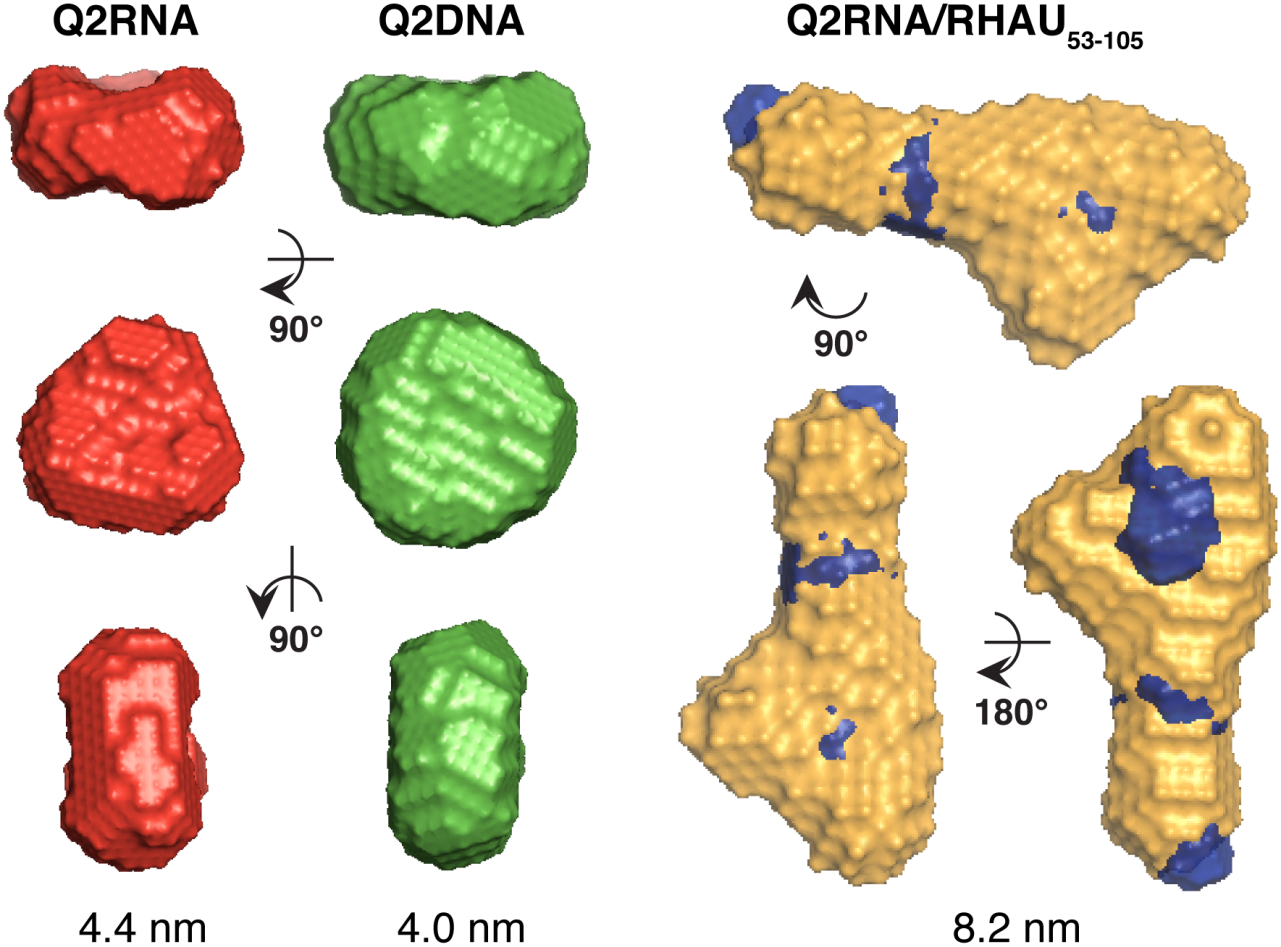
SAXS envelopes of Q2RNA, Q2DNA and Q2RNA/RHAU_53-105_ complex. Color code: Red, Q2RNA; green, Q2DNA; golden, Q2RNA/RHAU_53-105_ complex. *D*_*max*_ values are shown beneath each species. The SAXS shape model of RHAU_53-105_ (blue) was superimposed on the complex.

SAXS data for Q2RNA, Q2DNA AND Q2RNA/RHAU_53-105_ complex collected at multiple concentrations were merged to obtain a single scattering profile (Fig 7C). The pair distance distribution function, P(r), which represents a histogram of all observed distances between electron pairs in the molecule was obtained from merged data using program GNOM (Fig 7D). Both Q2RNA and Q2DNA demonstrate a P(r) plot consistent with a globular structure, whereas the Q2RNA/RHAU_53-105_ complex likely adopts an extended conformation based on the elongated tail at longer distances. From this analysis, the radius of gyration (*r*_*G*_) and maximum particle dimension (*D*_*max*_) were determined (Table 1), and used as constraints to generate 20 individual low-resolution models. Individual models, with the chi (χ) values shown in Table 1, were rotated and superimposed to obtain an averaged solution conformation (Fig 8). Excellent superimposition of individually calculated models were confirmed by the normalized spatial discrepancy (NSD) parameter (≤ 0.63) for each molecule ensemble. Both RNA and DNA G4s adopt disc-shaped structures with concave bevels at the top and bottom, while the Q2RNA/RHAU_53-105_ complex adopts an extended shape. Superposition of the previously determined RHAU_53-105_ solution structure by SAXS onto the Q2RNA/RHAU_53-105_ complex suggests that G4 recognition is occurring via one of the termini of the protein.

### Amino acids in and adjacent to the RSM mediate recognition of RNA G4

**Fig 9:**
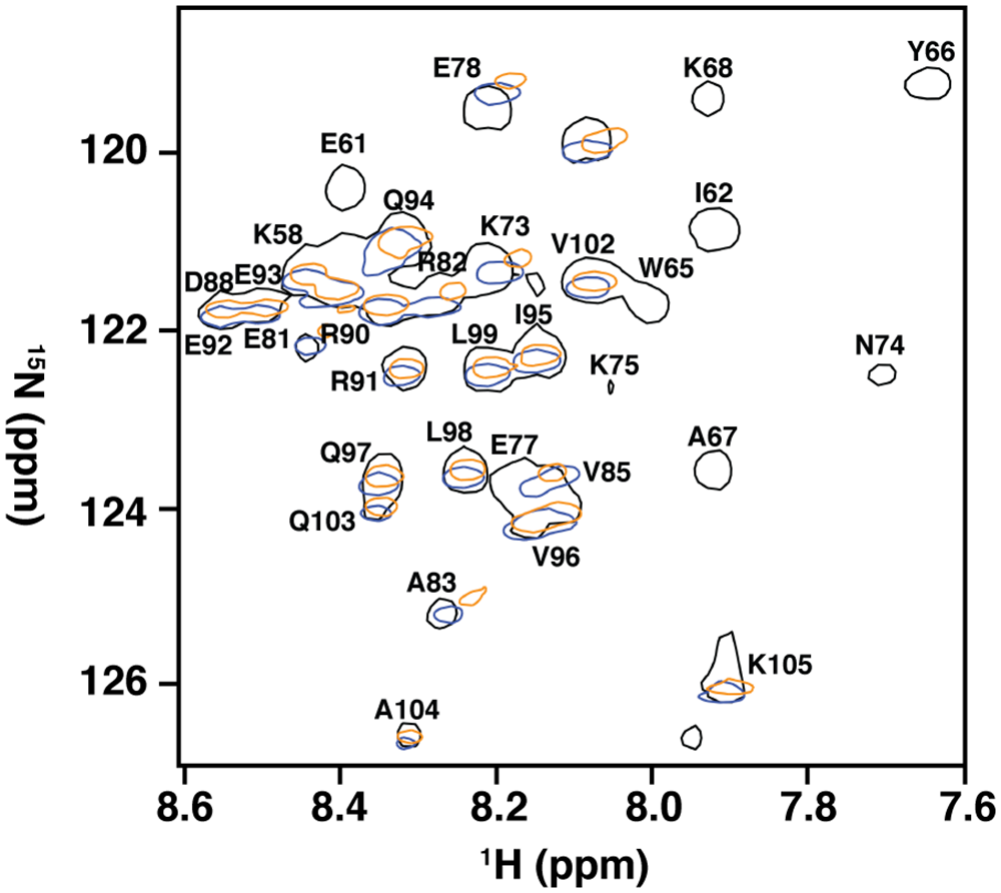
Amino acids in and adjacent to the RSM mediate recognition of RNA G4. ^15^N-HSQC spectral overlay of RHAU_53-105_ free (black) and in complex with Q2RNA (orange) or hTR_1-20_ (blue) with a subset of assigned resonances labeled. Data for RHAU_53-105_ and RHAU_53-105_/hTR_1-20_ were previously obtained, and used for comparison (30).

To determine the region of RHAU_53-105_ involved in mediating the interaction with G4, we expressed and purified isotopically-enriched ^15^N-RHAU_53-105_. After equimolar mixture with Q2RNA, we successfully purified Q2RNA/^15^N-RHAU_53-105_ complex, and acquired its ^15^N- HSQC spectrum (Fig 9). To determine the region(s) perturbed by G4 binding, we compared the determined HSQC spectrum to those previously determined for free ^15^N-RHAU_53-105_ and ^15^N-RHAU_53-105_ in complex with another minimal RNA G4 (hTR_1-20_) [31]. We observed nearly identical chemical shift perturbations as previously reported, with significant chemical shift perturbations clustering to the RSM and adjacent α-helix (K58, R60, E61, I62, G63, M64, W65, Y66, A67, K68, K69, N74, K75, A77, and E78). Therefore, we conclude that, as previously observed, residues in the RSM and the adjacent helix are responsible for mediating the G4 interaction.

## Discussion

G4s were predicted to play key roles in a number of biological activities including the regulation of gene transcription and translation [10], and evidence for that has accumulated in recent years both *in vitro* and *in vivo* [15, 58, 59]. Various proteins interact specifically with G4s, suggesting they fulfill important functions in cellular processes [60]. RHAU has been observed in a number of contexts to interact with RNA G4s, but the mechanism of how it recognizes and unwinds these structures is not well characterized. We have chosen as our model system a specific G4 (Q2RNA) found in the 3’-UTR of the PITX1 mRNA, primarily because its interaction with RHAU in a cellular context is established [26]. Based on several methods, including staining with an orientation-specific dye, TDS, and spectropolarimetry, Q2RNA is a parallel G4, and adopts a compact, disc-shaped conformation in solution that is consistent with another previously determined RNA G4 structure by SAXS [31]. Interestingly, the DNA equivalent (Q2DNA) presented markedly different features, namely in terms of its CD profile and its inability to stain with a parallel G4 dye, despite adopting a similar shape in solution as determined by SAXS and sharing similar hydrodynamic features as Q2RNA. Although a high-resolution structure would unambiguously highlight the differences, our data is consistent with Q2DNA adopting the hybrid-type G4 orientation observed in group II G4s, which has three parallel and one antiparallel strands [55–57]. We anticipate that the ability to accommodate 2′-OH groups in RNA G4s (affecting hydrogen bonding and sugar puckering) is central to the observed conformational differences between Q2RNA and Q2DNA [61].

Previous low-resolution structural and biophysical studies suggest that the N-terminal domain of RHAU interacts with the G-quartet face on the top or bottom plane of the G4 for both RNA and DNA [31]. Recently, a high-resolution structure of a short N-terminal peptide in complex with a DNA G4 has reinforced this mode of recognition, but also suggested that certain basic amino acid residues mediate specificity through interaction with the phosphodiester backbone [30]. Recognition of the Q2RNA G4 by the N-terminal region of RHAU (containing the RSM) uses nearly identical amino acid residues to those previously observed as important with another RNA G4 [31]. The elongated solution structure of Q2RNA/RHAU_53-105_ by SAXS is consistent with the same protein truncation in complex with another RNA G4 [31], and the superimposition of individual Q2RNA and RHAU_53-105_ models onto the complex model are consistent with recognition of the G-quartet face as the primary site of recognition.

Mechanistic studies have also suggested the importance of a parallel orientation for the recognition by RHAU [24, 30]. The parallel G4 specific dye used in this study (NMM) interacts by stacking on the G-tetrad faces [53] and we have observed a significant reduction in the staining intensity of the dye where the G4 is bound to RHAU_53-105_ as opposed to free G4 (data not shown). This suggests that the protein occupies the tetrad face. A previous study investigating a G4 from the human telomerase RNA (hTR) and its DNA counterpart has revealed that both adopt a parallel orientation and that both interact with RHAU_53-105_ by means of the RSM. However, the DNA G4 made additional interactions with RHAU that were not observed in the RNA G4 [31]. DNA G4s generally demonstrate lower affinity for RHAU than their RNA counterparts [28, 30, 31], and whether the 2’-OH, a parallel arrangement, or both are important remains to be determined for RNA binding. Given these observations, it was not surprising that different strand orientations adopted by Q2RNA and Q2DNA significantly impact their affinity for RHAU. In the absence of a high-resolution RHAU-RNA G4 structure, our results strongly support the previously observed mode of recognition where strand directionality is key to presenting a parallel G4 face for RHAU binding. High-resolution structural studies of RNA G4s in complex with RHAU will likely confirm the hypothesis that both electrostatic and steric impacts of the 2’-OH also fulfill an important role.

While the importance of the N-terminal domain of RHAU has clearly been established, the mechanism whereby full-length protein binds and unwinds G4 structures remains to be elucidated. Binding of truncated RHAU_53-105_ to RNA or DNA G4 does not attain the full binding affinity observed in full-length RHAU nor does it induce unwinding [27, 28, 31]. These features are clearly confirmed again in this study as full-length RHAU has higher affinity than the N-terminal fragment for Q2RNA, and comparison of the CD spectra of Q2RNA free and in complex with RHAU_53-105_ indicates no G4 unwinding. Therefore, future studies geared towards an understanding of G4 helicase activity in the context of the full-length protein, remain a priority. The work presented here, while focused specifically on the *in vitro* study of a purified RNA-protein complex, provide the template for an eventual mechanistic understanding of G4 impact on translational regulation of mRNAs, including PITX1.
